# The effect of auditory rhythm on the temporal allocation of visual attention in aging

**DOI:** 10.3389/fpsyg.2025.1529967

**Published:** 2025-02-11

**Authors:** Zhihan Xu, Juan Huang, Yuxuan Shen, Yanna Ren, Yulin Gao, Ting Guo

**Affiliations:** ^1^Department of Foreign Language, Ningbo University of Technology, Ningbo, China; ^2^Department of Psychology, College of Humanities and Management, Guizhou University of Traditional Chinese Medicine, Guiyang, China; ^3^Department of Psychology, Jilin University, Changchun, China

**Keywords:** rhythm, temporal expectation, aging, cross-modal, tempo

## Abstract

**Introduction:**

Isochronous rhythm has been shown to induce temporal expectation, allocated attention to specific points in time to optimize behavioral performance, both within a single modality and across different modalities, in younger adults. However, it remains unclear how an isochronous rhythm in one modality influences the temporal allocation of attention in another modality among older adults. Moreover, whether the cross-modal temporal expectation effect in aging is influenced by tempo has not yet been explored.

**Methods:**

To address these issues, both younger and older participants performed a rhythmic temporal expectation task in which auditory isochronous rhythms, presented at either 600 ms (faster) or 1,400 ms (slower) tempo, were used to trigger temporal expectation for a visual target.

**Results:**

The results demonstrated a cross-modal temporal expectation effect, with participants exhibiting significantly faster responses when the visual target appeared in synchrony with the preceding auditory rhythm compared to out-of-synchrony trials. This effect was evident in both younger and older groups and was not influenced by tempo.

**Discussion:**

These findings suggest that the ability to utilize auditory isochronous rhythms to drive the temporal allocation of visual attention can be preserved in normal aging, highlighting the robustness of cross-modal temporal expectations across both younger and older adults.

## 1 Introduction

The brain is recognized as a predictive organ, continuously generating expectations about what we will hear, see, or touch. These predictions play a crucial role, essential for responding effectively to our dynamic, multifaceted surroundings. At the core of these predictive processes is the capacity to allocate attention to specific time points to enhance behavioral performance, a process referred to as temporal expectation (Coull and Nobre, 1998; Coull and Nobre, 2008; Nobre and Van Ede, 2018). Temporal expectation is a fundamental survival skill. For example, a musical beat helps us anticipate the onset of the next beat, so we can sing in sync with the rhythm, or the temporal parameters of a suddenly incoming ball’s trajectory help us avoid being hit (Nobre et al., 2007).

Numerous studies have suggested that temporal expectations could be triggered by isochronous rhythms. When participants engaged with a temporally regular stimulus structure, they automatically adapted to it, aligning their internal oscillatory patterns of attentional pulse peaks and troughs with the rhythmic stream (Large and Jones, 1999; Large, 2000; McAuley and Jones, 2003). When the target occurred in synchrony with the preceding rhythmic pace, participants demonstrated faster and better behavioral performance compared to when it appeared out of synchrony (Barnes and Jones, 2000, Jones et al., 2006; Lange, 2010; Jones et al., 2017; Ren et al., 2019).

Although the effectiveness of isochronous rhythm in temporal expectation has been demonstrated in both auditory (Jones et al., 2002; Bolger et al., 2014; Cutanda et al., 2015) and visual modalities (Correa and Nobre, 2008; Rohenkohl and Nobre, 2011; Trivino et al., 2011; Breska and Deouell, 2014), as well as in cross-modal paradigms (Miller et al., 2013), the growing literature in this area has predominantly focused on younger adults (Rohenkohl et al., 2011; Sanabria and Correa, 2013; Breska and Deouell, 2016; Breska and Deouell, 2017; Jones et al., 2017; Xu et al., 2021a). Fewer studies have examined older adults, and those that have investigated the relationship between aging and rhythmic temporal expectation have been limited to a single modality (Gallego Hiroyasu and Yotsumoto, 2020; Xu et al., 2024). However, current research on symbolic cue-based temporal expectation in aging includes studies involving both single modality and cross-modal designs.

Temporal expectation could be driven not only by isochronous rhythms but also by symbolic cues (Coull and Nobre, 2008). Zanto et al. (2011) proposed that older adults have impairments in using symbolic cues to form temporal predictions. They employed a temporal cueing task using visual cues, where the letters S and L served as predictive cues, indicating that a visual target would appear after 600 ms or 1,400 ms, respectively, while the letter N acted as a neutral cue providing no information about the target’s onset time. They found that younger adults responded much faster to predictive cues compared to neutral cues, demonstrating that they could effectively use symbolic cues to accelerate their responses. In contrast, older adults did not exhibit a similar effect, suggesting that they were unable to use these symbolic cues to form temporal expectations (Zanto et al., 2011). Chauvin et al. (2016) conducted a similar experiment and found that cue-based temporal expectation is preserved in normal aging. In contrast to the Zanto and colleagues’study, their experiment used auditory cues, specifically high-frequency (1,600 Hz) and low-frequency (400 Hz) tones, signaling the onset of a visual target following a short or long temporal interval, respectively, while a neutral beep (1,000 Hz) provided no information about the target’s onset time. Their findings revealed that both younger and older adults were able to effectively use the auditory cues to improve performance for visual targets, showing no significant age-related differences (Chauvin et al., 2016).

However, to date, no researchers have specifically examined how an isochronous rhythm in one modality influences the temporal allocation of attention in another modality among older adults. Since human experience is inherently multisensory and, in daily life, we frequently shift attention between different sensory modalities, it is crucial to explore how these modalities interact. Prior research has demonstrated that sensory modalities play a crucial part in age-related changes in attention (Guerreiro et al., 2010). Therefore, cross-modal analysis in older adults can reveal how different sensory systems collaborate within this group and, compared to unimodal modality, provide a more comprehensive understanding of temporal perception in real-world contexts.

In addition, previous research on temporal expectation has typically been driven by isochronous rhythms with a faster tempo, specifically referring to the inter-onset intervals (IOIs) between two consecutive stimuli within the rhythmic sequence, which is usually less than 1 s (Lange, 2009; Sanabria et al., 2011; De la Rosa et al., 2012). However, the evidence from neuropsychological studies suggests distinct mechanisms underlying the temporal processing of intervals above and below 1 s (Lewis and Miall, 2003a,b; Coull et al., 2011). Moreover, Xu et al. (2021a) also proposed that the mechanism underlying rhythmic temporal expectation differs depending on the tempo, with the processing shifting from automatic to requiring more attentional resources as the tempo slows down (Xu et al., 2021b). Additionally, developmental studies on preferred tempo (referring to the rate of a series of sounds perceived as neither too fast nor too slow) have shown that the preferred tempo for older adults is slower than that for younger adults (Drake et al., 2000, Vanneste and Wearden, 2001; Jones et al., 2006). Therefore, whether the cross-modal temporal expectation effect in older adults is influenced by tempo remains unexplored.

In summary, the primary aim of our present study is to investigate whether older adults have a deficit in cross-modal rhythmic temporal expectation. A secondary aim is to examine the impact of tempo on the cross-modal temporal expectation effect. To clarify these issues, we adopted a rhythmic temporal expectation task, using auditory isochronous rhythm to drive the temporal allocation of attention to visual target. Additionally, we involved two tempos: 600 ms representing the faster tempo and 1,400 ms representing the slower tempo. This approach allowed us to investigate the cross-modal rhythmic temporal expectation effect in both younger and older adults, across varying tempos, from milliseconds to seconds.

## 2 Methods

### 2.1 Participants

The study comprised 36 participants, divided into two groups: 18 younger adults (8 females, ages 19–20 years, mean age = 19.11, SD = 0.32) and 18 older adults (10 females, ages 65–75 years, mean age = 68.78, SD = 3.21). The younger participants were engaged from Ningbo University of Technology, the older adults were sourced from the surrounding community. All participants self-reported being right-handed, with normal or corrected-to-normal vision and hearing. None were on psychotropic or vasoactive medication, nor did they have any history of psychiatric or neurological disorders. Additionally, none of the participants had undergone professional music training or played an instrument. The study received approval from the institutional ethics committee, and all participants provided written informed consent for their participation. The Mini Mental State Examination (MMSE) (Folstein et al., 1975) was conducted to participants to exclude individuals with dementia. All participants achieved the threshold score of 27 (mean score = 29.44, SD = 0.7).

### 2.2 Apparatus and stimuli

Participants sat comfortably in a dimly lit, soundproof chamber, positioned 60 cm away from a 27-inch monitor screen (1920 × 1,080 resolution, 60 Hz refresh rate). Stimuli presentation and reaction times (RT) collection were controlled by E-prime 3.0 software (Schneider et al., 2002).

All visual stimuli were presented centrally on the computer screen with a black background. The fixation point, represented as a white cross with a visual angle of 0.6° × 0.6°, was maintained visible throughout the experiment. The target comprised a white circle, measuring 4.5 cm in diameter (4.3° visual angle), presented for a duration of 150 ms. The auditory rhythm consisted of an isochronous sequence of five 700 Hz pure tones, each lasting 100 ms, with fixed interonset intervals (IOIs) of either 600 ms for the faster tempo or 1,400 ms for the slower tempo. These auditory stimuli were emitted through two loudspeakers, positioned 50 cm to the left front and right front of the participants, at a comfortable listening level.

### 2.3 Procedure

**Figure 1:**
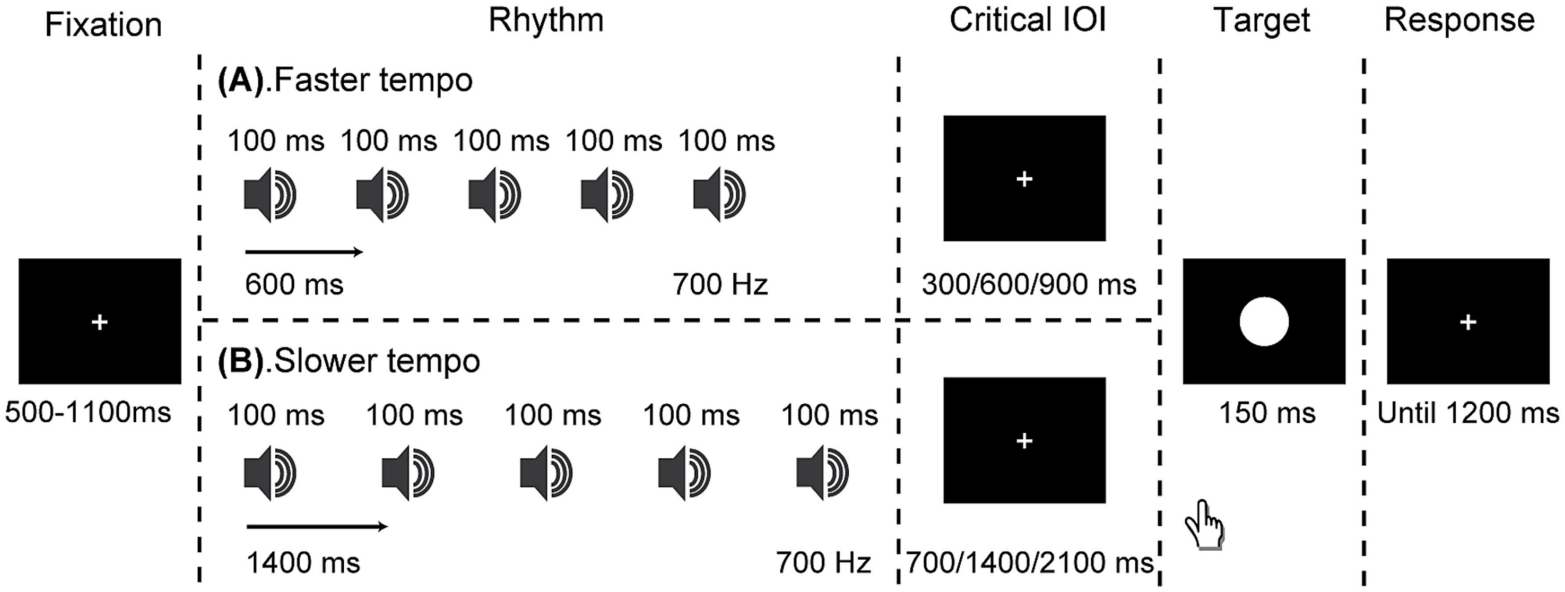
Schematic representation of events in a trial. Participants were instructed to respond to a white circle target that appeared either in-synchrony or out-of-synchrony with the rhythm, which was presented at a faster (600 ms; panel **A**) or slower (1,400 ms; panel **B**) tempo.

Each trial began with the fixation point being displayed for a variable duration of 500–1,100 ms (refer to Figure 1). Following this, the auditory isochronous rhythm was played at either a faster tempo of 600 ms or a slower tempo of 1,400 ms. Once the final tone of the auditory rhythm was played, the rhythm sequence ended, immediately followed by the appearance of the white circle target, which was presented either in-synchrony or out-of-synchrony (early or late) with the rhythm. Specifically, the onset of the target occurred after the rhythm at three critical IOIs: in synchrony (faster: 600 ms; slower: 1400 ms), early (faster: 300 ms; slower: 700 ms), and late (faster: 900 ms; slower: 2100 ms). Participants received both written and oral instructions to react to the target’s appearance as fast as possible, using their right index finger to press the left button of the mouse, and being cautious to avoid premature responses before the target appeared. A response time window of up to 1,200 ms was allowed.

### 2.4 Design

The experiment employed a mixed factorial design, incorporating a between-participants factor: age group (younger, older) and two within-participants factors: tempo (faster, slower), and time (early, in synchrony, late).

Each participant completed 6 experimental blocks, with 3 faster and 3 slower tempo blocks, each containing 40 trials. The arrangement of the tempos was counterbalanced across participants. Prior to each tempo experimental session, participants completed a training block with 20 practice trials. Across trials, the target occurred in synchrony with the rhythm preceding it in 60% of the trials and out of synchrony in 30% (early and late equiprobably). In the remaining 10% of trials, there was no target stimulus in these trials; these were designated as catch trials. Catch trials were employed to counteract the impact of a “hazard function,” where expectations were created based on the conditional probability that the stimulus would occur given that it had not yet appeared (Correa et al., 2006).

### 2.5 Data analysis

RT was calculated as the interval between the target’s onset and the participant’s motor response. Training and catch trials were excluded from the analysis. Additionally, responses made before the target’s appearance (anticipatory responses), trials where no response was made after the target’s appearance (omission errors), and trials with RTs exceeding three standard deviations from an individual’s average RT for each dependent variable were omitted. A total of 232 trials were excluded across the 18 younger participants, and 189 trials were excluded across the 18 older participants, based on these criteria. According to the results of the two-sample *t*-tests: *t* (34) = −0.847, *p* = 0.405, there was no significant difference between the two groups.

A 3-way mixed-design analysis of variance (ANOVA) was conducted using SPSS version 19.0 (SPSS, Tokyo, Japan) to examine the effects of the between-participants factor (age group: younger, older) and the two within-participants factors (tempo: faster, slower; time: early, in synchrony, late). Greenhouse–Geisser corrections were applied to address potential sphericity violations, with adjustments made to degrees of freedom as necessary. Statistical significance was determined at *p* < 0.05, and effect sizes (*η*_p_^2^) estimates were also reported.

## 3 Results

The detailed mean RTs for each condition are provided in Table 1. A 3-way mixed-design ANOVA, considering the factors of tempo (faster, slower), time (early, in synchrony, late), and age group (younger, older), identified a significant main effect of age [*F* (1,34) = 9.926, *p* = 0.003, *η*_p_^2^ = 0.226], indicating that younger participants exhibited faster RTs (234.42 ms) compared to older participants (273.2 ms). There was also a significant main effect of tempo [*F* (1,34) = 135.588, *p* < 0.001, *η*_p_^2^ = 0.8], with participants responding faster in the faster tempo (237.67 ms) than the slower tempo condition (269.95 ms).

**Table 1:** Mean RTs (ms) for each Group (younger, older), Tempo (faster, slower) and Time condition (early, in synchrony, late).

	Younger group		Older group	
	Faster tempo	Slower tempo	Faster tempo	Slower tempo
Early	242 (11)	281 (17)	277 (11)	325 (20)
In synchrony	188 (3)	204 (4)	228 (10)	245 (11)
Late	226 (12)	265 (10)	264 (10)	301 (13)

Values in parentheses are standard errors of the mean.

**Figure 2:**
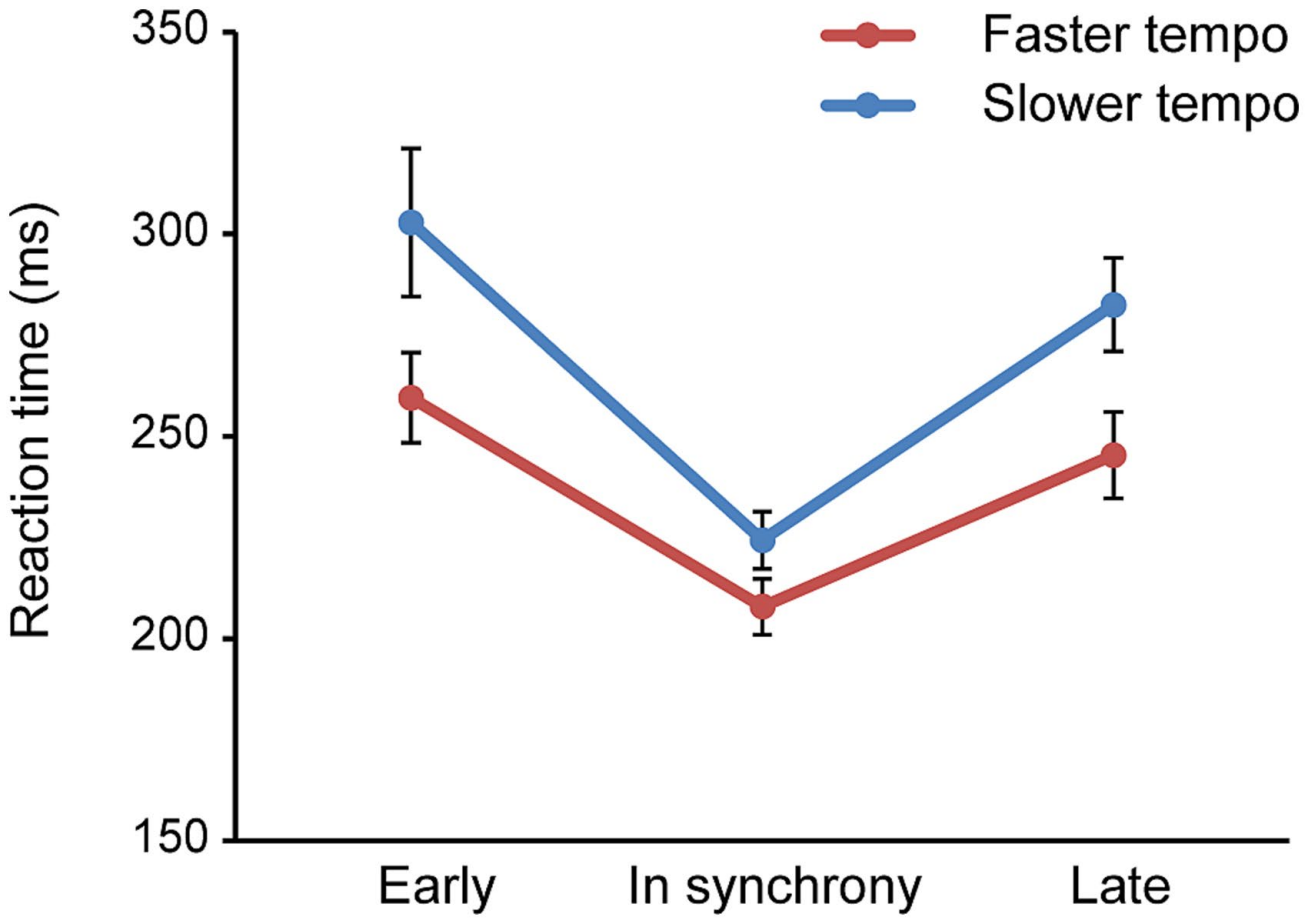
Mean RT plotted against the time condition (early, in synchrony, late) for faster and slower tempo, collapsed across age groups. Error bars reflect the standard error of the mean.

Additionally, analysis revealed a significant main effect of time [*F* (2,68) = 32.177, *p* < 0.001, *η*_p_^2^ = 0.486], with RT curves being U-shaped across all conditions. Specifically, RT reached its lowest point when the target was presented in synchrony with the rhythm preceding it, before subsequently increasing again. Further pairwise comparisons demonstrated the fastest RT (216.18 ms) occurred in synchrony, compared to when the target appeared out of synchrony, either earlier (281.26 ms) or later (263.99 ms) (in synchrony < early, late; both *p* < 0.001), reflecting the effect of temporal expectation (Figure 2). A significant interaction between tempo and time was also observed [*F* (2,68) = 5.894, *p* = 0.012, *η*_p_^2^ = 0.148], indicating that the U-shaped curves were sharper in the slower tempo condition compared to the faster tempo condition.

**Figure 3:**
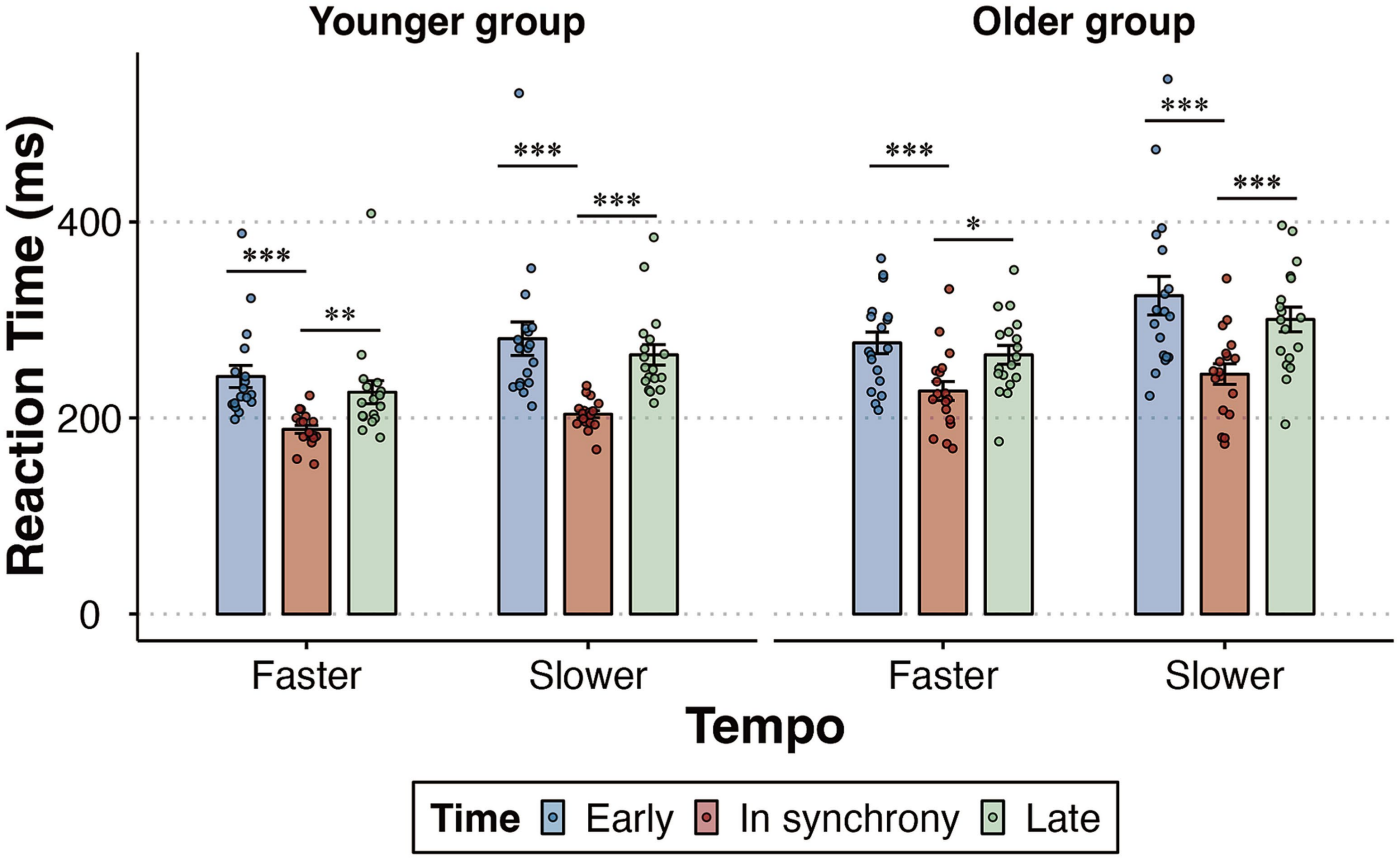
Mean RT as a function of the tempo (faster, slower) and time (early, in synchrony, late) condition among younger and older groups. Error bars reflect the standard error of the mean. Statistical significance levels are marked as ***p < 0.001, ** *p* < 0.01, * *p* < 0.05.

The key finding was that both younger and older participants exhibited significant effects of rhythm-driven temporal expectations, improving the speed of target detection. Follow-up pairwise comparisons, adjusted using the Bonferroni correction, revealed that under both faster and slower tempo conditions, participants responded faster when targets were presented in synchrony with the rhythm than when they were presented at the early or late moments in both younger (faster: both *p* ≤ 0.009; slower: both *p* < 0.001) and older participants (faster: both *p* ≤ 0.012; slower: both *p* < 0.001) (Figure 3). The time × age interaction [*F* (2,68) = 0.016, *p* = 0.97, *η*_p_^2^ = 0.0005] and tempo × time × age interaction [*F* (2,68) = 0.252, *p* = 0.693, *η*_p_^2^ = 0.007] were not significant.

**Figure 4:**
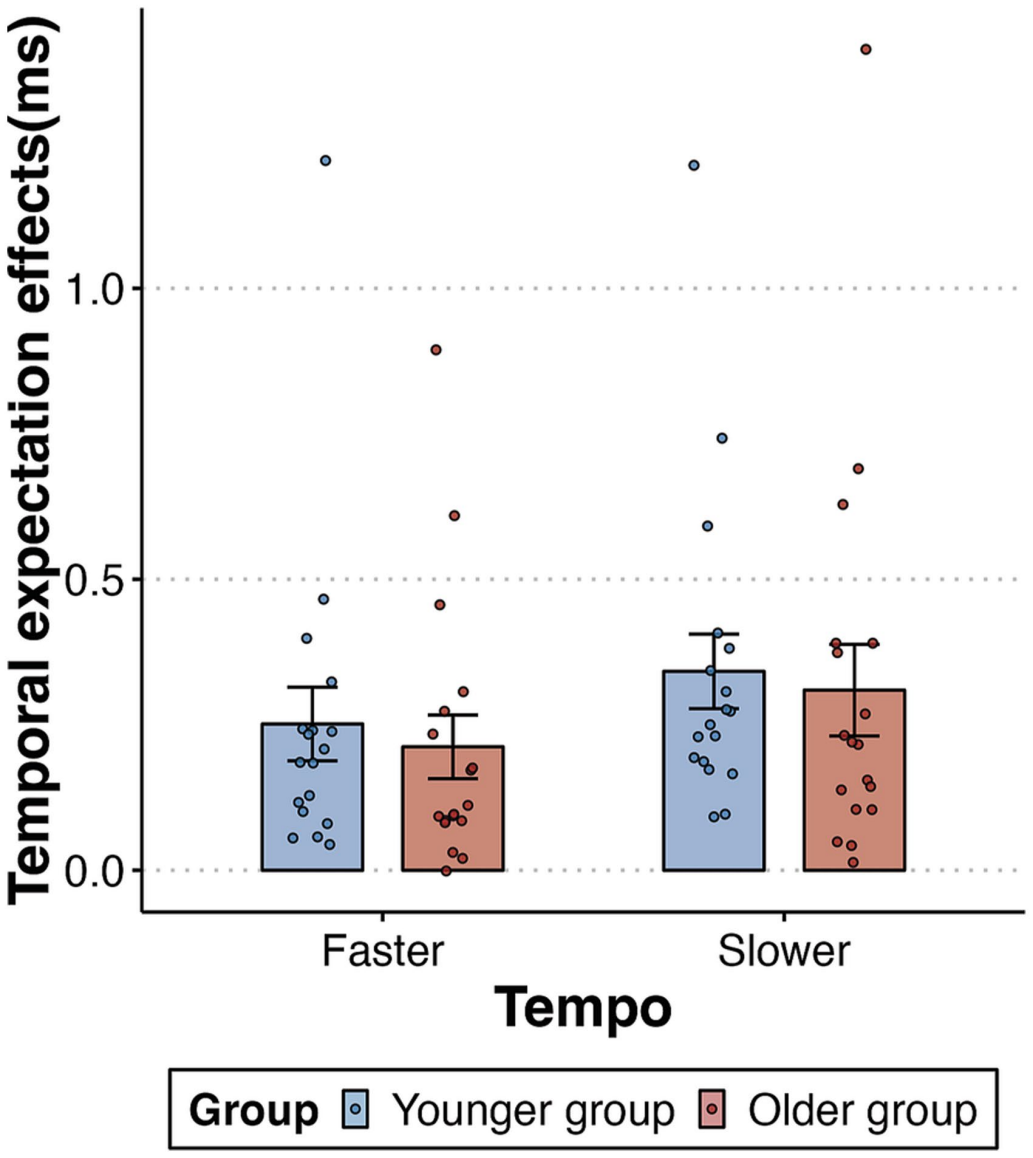
Temporal expectation effects (out-of-synchrony RT minus the in-synchrony RT, divided by in-synchrony RT) as a function of tempo (faster and slower) and group (younger and older). Error bars reflect the standard error of the mean.

To rule out the influence of older participants’ generally slower responses, we conducted a further analysis of the data focusing on the temporal expectation effect, instead of the mean RTs. This effect was quantified by calculating the mean RT difference between out-of-synchrony trials (collapsing early and late trials) and in-synchrony trials, divided by the mean RT in the in-synchrony trials. A 2-way mixed-design ANOVA for 2 tempo (faster, slower) and 2 age group (younger, older) demonstrated a significant main effect of tempo [*F* (1,34) = 18.025, *p* < 0.001, *η*_p_^2^ = 0.346]. We found no evidence of either a main effect of age group [*F* (1,34) = 0.162, *p* = 0.69, *η*_p_^2^ = 0.005] or an interaction between tempo and age [*F* (1,34) = 0.019, *p* = 0.89, *η*_p_^2^ = 0.001] (Figure 4). In summary, both older and younger adults demonstrated significant and equivalent effects in using auditory isochronous rhythms to guide the temporal allocation of visual attention.

## 4 Discussion

In the present study, we used a rhythmic temporal expectation task to investigate the impact of age on cross-modal rhythmic temporal expectation, specifically examining how auditory rhythm influences the temporal allocation of visual attention in aging. The results revealed that both older and younger adults exhibited cross-modal temporal expectation effects under both faster and slower tempo conditions, with faster reaction times when the visual target was presented in synchrony with the auditory rhythm preceding it compared to when it appeared early or late.

Consistent with previous research, we found that as the tempo slowed, reaction times increased. This may be due to the fact that the range within which beats are perceived is limited, typically falling between approximately 100 ms and 2.5 s (Friberg and Sundström, 2002; London, 2012). It has been suggested that the preferred tempo (neither too fast nor too slow) for younger adults is approximately 600 ms, whereas it is closer to 700 ms for older adults (Drake et al., 2000; McAuley et al., 2006). When the tempo is too fast, successive sounds become difficult to distinguish; when the tempo is too slow, rhythmic structure is likely to break down, resulting in a sequence of isolated sounds (Fraisse, 1963; Fraisse, 1982). In addition, our previous research has indicated that temporal expectations formed by slower tempo may require more deliberate processing and rely on memory-based strategies compared to those formed by faster tempo (Xu et al., 2021a). Moreover, automatically processed temporal expectations have been found to enhance behavioral performance more effectively than those relying on memory-based strategies (Ren et al., 2019; Xu et al., 2021b). Consequently, the results of the present study showed that both younger and older adults achieved faster reaction times in the 600 ms faster tempo condition compared to the slower 1,400 ms tempo condition.

Moreover, we observed that the reaction times, especially for older adults, were faster than those reported in previous studies. We speculate that part of the reason for this inconsistency might be the isochronous rhythm, which helped older adults predict when the target would appear, thus accelerating response times. Therefore, in studies reporting that older adults benefit from temporal information to form temporal expectations, reaction times were generally faster than in studies reporting temporal expectation deficits in older adults (Zanto et al., 2011; Chauvin et al., 2016; Xu et al., 2024). Additionally, we noted that, despite similar findings in studies showing that older adults retain temporal prediction abilities, reaction times for older adults in our study were still faster than those observed in previous research. However, as Chauvin et al. (2016) found, older adults can use symbolic cues to effectively allocate attention to specific time points (Chauvin et al., 2016). Differing from their study, we used isochronous rhythm. Isochronous rhythm has been suggested to be more effective in optimizing behavioral performance, providing a more precise attentional focus over time, compared to symbolic cues (Breska and Deouell, 2017; Ren et al., 2019; Xu et al., 2021a). Thus, isochronous rhythm facilitated faster reactions, resulting in quicker response times in our study with older adults. In addition, compared to a study that used visual rhythm to drive temporal expectation, older adults in our study also exhibited faster reaction times (Xu et al., 2024). We speculate that this may be attributed to the fact that the auditory system appears to be more suited for processing sequential information, which may make auditory rhythm more efficient in triggering temporal expectation (Lustig and Meck, 2001; Repp and Penel, 2002; Xu et al., 2021b). Nevertheless, it is necessary and interesting to confirm these hypotheses in future studies.

The current findings that younger adults can allocate visual focus on specific moments that align with predictions based on auditory isochronous rhythms, rather than to other time points, are consistent with previous reports (Miller et al., 2013). These results support the perspective of attention being a finite resource distributed across multiple modalities (Kahneman, 1973), which suggests that attentional entrainment in one modality can also facilitate the synchronization of attention within another modality, resulting in synchronized attentional peaks across both modalities rather than suppressing attention in the other. Furthermore, this study extends previous findings by demonstrating that the same cross-modal temporal expectation effect observed in younger adults is also preserved in older adults, highlighting the robustness of this effect across age groups.

Notably, we found no evidence of age-related decline in rhythmic temporal expectation under either faster or slower tempo conditions. This contrasts with a previous study that suggested the temporal expectations effect driven by isochronous rhythms in older adults could be influenced by tempo within a single visual modality. Specifically, they demonstrated that these expectations can be preserved in older adults at a tempo of 1800 ms; however, when the rhythm is faster, at 600 ms, age-related declines manifest (Gallego Hiroyasu and Yotsumoto, 2020; Xu et al., 2024). We infer that the difference may be attributed to the modality of the rhythm used; we employed an auditory rhythm, whereas previous research used a visual rhythm. Given that many auditory events (such as speech or music) unfold over time, as mentioned above, the auditory system has been suggested to be particularly adept at processing sequential information (Repp and Penel, 2002). Lustig and Meck (2001) also demonstrated that sensitivity to time is higher in the auditory modality compared to the visual modality, especially in older participants compared to younger ones (Lustig and Meck, 2001). As a result, older adults may be able to utilize auditory rhythms to form temporal predictions at faster tempos, but not visual rhythms. Another possibility lies in the cross-modal design of our task. In our current study, auditory rhythms triggered the visual temporal attention, whereas previous research relied on visual rhythms to drive the visual temporal attention. Cona et al. (2013) suggested that older adults take longer to disengage from visual stimuli, which raises the possibility that visual rhythms could potentially interfere with visual target processing (Cona et al., 2013). In contrast, auditory rhythms do not engage the visual system, and thus may cause less interference. Nonetheless, further research is needed to systematically verify these hypotheses.

## 5 Conclusion

In conclusion, the present study showed that both younger and older adults exhibited the fastest reaction times when the visual target appeared in synchrony with the preceding auditory rhythm, whereas reaction times were slower when the target appeared early or late. This finding suggested that both younger and older adults were able to use the auditory isochronous rhythm to trigger the temporal allocation of visual attention. Moreover, this cross-modal temporal expectation effect was not influenced by variations in tempo. These results highlighted the robustness of cross-modal temporal expectation mechanisms in both younger and older adults.

## Data Availability

The datasets presented in this study can be found in online repositories. The names of the repository/repositories and accession number(s) can be found at: https://osf.io/h8qem/.
